# Prevalence of Metabolic Syndrome and Its Determinants in Newly-Diagnosed Adult-Onset Diabetes in China: A Multi-Center, Cross-Sectional Survey

**DOI:** 10.3389/fendo.2019.00661

**Published:** 2019-10-01

**Authors:** Xia Li, Chuqing Cao, Xiaohan Tang, Xiang Yan, Houde Zhou, Jing Liu, Linong Ji, Xilin Yang, Zhiguang Zhou

**Affiliations:** ^1^Department of Metabolism & Endocrinology, The Second Xiangya Hospital, Central South University, Changsha, China; ^2^Key Laboratory of Diabetes Immunology, Central South University, Ministry of Education, Changsha, China; ^3^National Clinical Research Center for Metabolic Diseases, Changsha, China; ^4^Hunan Key Laboratory for Metabolic Bone Disease, Changsha, China; ^5^Department of Metabolism & Endocrinology, Gansu Provincial Hospital, Lanzhou, China; ^6^Department of Metabolism & Endocrinology, Peking University People's Hospital, Beijing, China; ^7^Department of Epidemiology and Biostatistics, School of Public Health, Tianjin Medical University, Tianjin, China

**Keywords:** type 2 diabetes mellitus, latent autoimmune diabetes in adults, type 1 diabetes mellitus, metabolic syndrome, insulin resistance, prevalence

## Abstract

**Aim:** The study aimed to investigate the prevalence of metabolic syndrome (MetS) and its determinants in newly-diagnosed adult-onset diabetes in China.

**Methods:** From April 2015 to October 2017, 15,492 consecutive patients with diabetes diagnosed within 1 year and aged ≥30 years were recruited from 46 tertiary care hospitals in 24 cities across China. Glutamic acid decarboxylase autoantibody was assayed centrally and clinical data were collected locally. Classic type 1 diabetes mellitus (T1DM), latent autoimmune diabetes in adults (LADA) and type 2 diabetes mellitus (T2DM) were defined using the criteria of American Diabetes Association, Immunology of Diabetes Society and World Health Organization. MetS was defined using Chinese Diabetes Society's criteria. Logistic regression analysis was used to obtain odds ratios (OR) of determinants of MetS.

**Results:** The overall prevalence of MetS was 66.5%, with the highest prevalence in T2DM (68.1%), followed by those in LADA (44.3%) and T1DM (34.2%) (*P* < 0.05 for all comparisons). After adjustment for traditional risk factors, T2DM had a 2.8-fold [95% confidence interval (CI): 2.36–3.37] MetS risk compared with LADA, whereas T1DM had significantly lower OR than LADA (OR: 0.68, 95% CI: 0.50–0.92). After further adjustment for insulin resistance, the OR of T2DM vs. LADA was slightly reduced but the OR of T1DM vs. LADA was greatly attenuated to non-significance (OR: 0.96, 95% CI: 0.70–1.33). In addition to types of diabetes, age, gender, geographical residence, education attainment, alcohol consumption and HOMA2-IR were independent determinants of MetS.

**Conclusions:** MetS was highly prevalent, not only in T2DM but also in T1DM and LADA in Chinese newly diagnosed patients; higher risk of MetS in LADA than in T1DM was partially attributable to higher insulin resistance in LADA.

## Introduction

Metabolic syndrome (MetS) is a constellation of cardiometabolic risk factors, the core components of which involve insulin resistance, abdominal obesity, hypertension, hyperglycemia, and dyslipidemia. In the general population, MetS increases the risks of cardiovascular disease (CVD), type 2 diabetes mellitus (T2DM) and all-cause mortality (1). Reassuringly, modification of MetS severity is associated with a concomitant reduction in the risk of further T2DM and CVD (2). So, identifying patients with MetS is critical for precise intervention targeting insulin resistance and CVD protection. It is well-known that MetS is prevalent in type 2 diabetes, but the exact status of MetS in different types of diabetes and its determinants remain undefined.

Autoimmune diabetes results from immune-mediated destruction of pancreatic insulin-producing β cells and subsequent insulin deficiency. The two subtypes of autoimmune diabetes, classic type 1 diabetes mellitus (T1DM) and latent autoimmune diabetes in adults (LADA), are of great clinical heterogeneity and thus different treatment regimes. While T1DM is characterized by its acute onset and life-long dependence on exogenous insulin, LADA is a slowly progressive form of T1DM, characterized by adult onset, presence of circulating islet autoantibodies, and insulin independence at least within 6 months after diagnosis of diabetes (3). Though not requiring insulin therapy initially, patients with LADA exhibit pronounced impairment in the maximally stimulated β-cell secretory capacity compared with T2DM patients (4), and β-cell secretory capacity in LADA deteriorates over time at a 3-fold higher rate than in T2DM (5). Generally, LADA lies in between T1DM and T2DM regarding autoimmune, inflammatory, metabolic and genetic features, often described as “end of the rainbow” (6). T1DM, LADA and T2DM jointly constitute a continuum of varying degrees of clinical features and β-cell function.

The presence of MetS in diabetes is a strong indicator for increased insulin resistance, which is common in T2DM (7). Growing evidence suggests that insulin resistance predisposes to accelerated β cell loss, being involved in the pathogenesis of autoimmune diabetes (8). A study reported that in European patients with diabetes, the prevalence of MetS was quite high, 31.9% in T1DM, 41.9% in LADA and 88.8% in T2DM (9). Although there is still a lack of randomized controlled trials that addresses the benefits of lowering Mets rates in autoimmune diabetes, several randomized controlled trials and epidemiological studies showed the benefits of vigorous control of glycemia, lipids and blood pressure on mortality and coronary artery disease in T1DM patients (10, 11). In this connection, a heterogeneity of MetS susceptibility genes was observed across ethnicities (12). Moreover, Asian populations have a different distribution of glutamic acid decarboxylase autoantibody (GADA) titers and a lower adiposity than Caucasians (13, 14), and therefore, the prevalence and features of MetS in Asians may differ from those in Caucasians. Nevertheless, there is still a lack of large-scale multicenter studies to document the prevalence of MetS in the entire spectrum of diabetes in Asian populations, and whether risk factors for MetS in the general population, including age, smoking, alcohol consumption, residence and family history of diabetes, also applied to population with autoimmune diabetes remained unclear (15, 16).

We analyzed the data from a nationwide, multicenter, cross-sectional survey to investigate the prevalence of MetS and its determinants in Chinese patients with newly diagnosed diabetes.

## Methods

### Research Design and Participants

**Figure 1:**
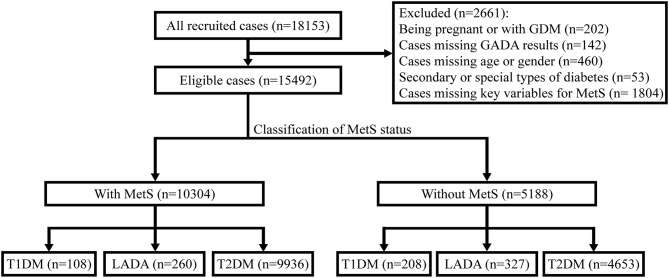
Flow diagram and classification of 15,492 newly-diagnosed adult-onset patients with diabetes for MetS status in China. GDM, gestational diabetes mellitus; GADA, glutamic acid decarboxylase autoantibody; MetS, metabolic syndrome; T1DM, type 1 diabetes mellitus; T2DM, type 2 diabetes mellitus; LADA, latent autoimmune diabetes in adults.

The present study was a nationwide, multi-center, cross-sectional survey conducted from April 2015 to October 2017. Patients were recruited consecutively from 46 tertiary care hospitals in 20 provincial administration areas and 4 municipalities, across the seven geographic regions of China (4 Northeast, 8 North, 3 Northwest, 9 Central, 3 Southwest, 7 South, and 12 East), thereby, representing the diversity in climates, cultures, and ethnicities of the Chinese populations. This survey was designed to collect detailed data on outpatients with newly-diagnosed diabetes, i.e., within 1 year of diabetes. The ethics review committee/institutional review board of each participating hospital approved the study, and the World Medical Association's Declaration of Helsinki was followed. Informed consent was obtained from all the participants before data collection. The inclusion criteria were as follows: (1) diagnosis of diabetes using the World Health Organization (WHO) 1999 criteria (17) and at ≥30 years of age; (2) diabetes duration < 1 year; and (3) outpatients attending metabolism and endocrinology clinics. The exclusion criteria were as follows: (1) pregnancy at diagnosis or gestational diabetes mellitus (GDM); (2) secondary or special type of diabetes; (3) presence of acute diseases that could interfere with the glucose metabolism; and (4) any malignancy. After excluding 1,804 patients whose data on key variables for diagnosis of MetS were missing, the remaining 15,492 patients were included for analysis (Figure 1).

### Classification of Diabetes

Diagnosis of T1DM was based on acute onset of insulin-requiring diabetes, proneness to ketosis or ketoacidosis, impaired insulin secretion or GADA positivity. LADA was diagnosed based on the Immunology of Diabetes Society (IDS) criteria (3): (1) GADA positivity; (2) diagnosed at ≥30 years of age; (3) insulin independence for at least 6 months postdiagnosis. GADA-negative patients not requiring immediate insulin therapy were diagnosed as with T2DM.

### Definition of Metabolic Syndrome

MetS was defined using the 2017 Chinese Diabetes Society's (CDS) criteria (18), and was diagnosed when three or more of the following criteria were met: (1) abdominal obesity: waist circumference ≥90 cm in men and ≥85 cm in women; (2) hyperglycemia: fasting blood glucose ≥6.1 mmol/L or 2-h blood glucose ≥7.8 mmol/L or previously diagnosed diabetes with treatment; (3) hypertension: blood pressure ≥130/85 mmHg or currently under antihypertension therapy; (4) fasting triglycerides (TGs) ≥1.70 mmol/L; (5) fasting high-density lipoprotein cholesterol (HDL-C) <1.04 mmol/L. All patients in this study were defined to fulfill the criteria for hyperglycemia. Lipid-lowering therapies were not included in the criteria.

### Estimation of Beta Cell Function and Insulin Resistance

An updated Homeostasis Model Assessment (HOMA2) calculator (University of Oxford, Oxford, UK) (19) was employed for estimation of beta cell function (HOMA2-B) and insulin resistance (HOMA2-IR), based on fasting plasma glucose and C-peptide concentrations.

### Data Collection Procedures

Demographic characteristics, medical history and lifestyle information were collected using a standard questionnaire via face-to-face interviews by uniformly-trained research nurses. Body height, weight, waist circumference, and blood pressure were measured by the research nurses. Medication information was retrieved from medical records. Smoking/drinking was classified as a binary variable assessed using a yes or no question depending on the current smoking/drinking status. Educational attainment was classified into three categories: “junior high school or lower,” “senior high school,” or “tertiary or above”. The Qinling Mountains and Huaihe River marked the boundary between North and South of China.

### Laboratory Assays

Fasting plasma glucose (FPG), TGs, HDL-C, LDL cholesterol (LDL-C), fasting C-peptide (FCP), and hemoglobin A1c (HbA1c) were assayed at the study sites by standard methods using overnight fasting venous blood samples. Postprandial blood samples were tested for 2-h postprandial plasma glucose (PPG) and C-peptide (PCP). Serum samples were transported to the core laboratory (Central South University) for antibody assays on ice within the day and stored at −80°C before analysis.

FCP and PCP were measured uniformly by the chemiluminescence method, but different kits were used in the participating centers. In Central South University, FCP and PCP were measured using Adiva Centaur Systema kit (Siemens, Munich, Germany).

GADA was measured by radioligand binding assays in duplicate, using *in vitro* transcribed and translated [^35^S] methionine-labeled recombinant human GAD65 (aa: 1–585) as previously described (13, 20). Briefly, 5 μL of human serum was incubated overnight at 4°C with 30,000 cpm of the ^35^S-GAD in a final volume of 200 μL in TBST buffer (50 mM Tris-HCl, 150 mM NaCl, 0.15% v/v Tween-20, pH 7.2). Subsequently, the immune complexes were isolated, washed, mixed with scintillation fluid (PerkinElmer, CT, USA) and radioactivity determined (Micro Beta Trilux 1450 counter; PerkinElmer, Finland). The cut-off value of ≥18 U/ml of WHO units, i.e., the 99th percentile of 405 healthy subjects, was used to define positivity for GADA. Further validations were performed to confirm the positivity. The sensitivity and specificity of the GADA assay were 82% and 97.8%, respectively, as assessed in the 2016 islet autoantibody standardization program (IASP 2016).

### Statistical Analysis

SPSS 20.0 software (IBM Corporation, Armonk, NY, USA) was used to perform all the data analysis unless specified. Normal distribution was checked using Kolmogorov-Smirnov test. Continuous data were presented as mean ± standard deviation (SD) if their normality was not rejected, or median (interquartile range) otherwise. Categorical variables were expressed as number (percentage). For variables with normal distribution, comparisons between groups were performed using independent Student *t*-test. Mann-Whitney *U* test was used to compare differences of variables whose normality was rejected. χ^2^ test or Fisher's exact test where appropriate was used to compare differences in categorical variables between groups. Effect sizes of comparisons between groups were calculated as previously reported (21, 22). After checking P-P plots of standardized regression residuals for normality, logistic regression analysis was performed to obtain odds ratios (ORs) and their 95% confidence intervals (CIs) of types of diabetes and other factors for MetS. A structured adjustment scheme was used to adjust for confounding effects of other variables. First, we performed univariable analysis. Second, we performed multivariable analysis with stepwise selection of confounders from traditional and potential MetS risk factors (*P* = 0.05 for entry and *P* = 0.10 for exit), including age, gender, region, education attainment, family history of diabetes, smoking and alcohol consumption habits. Third, we further adjusted for HOMA2-IR to check whether the increased or decreased risks of MetS in T2DM and T1DM was attributable to different levels of insulin resistance in those types of diabetes. A two-sided *P* < 0.05 was considered statistically significant.

## Results

### Characteristics of the Study Patients

The cohort had 52.6 (SD: 11.5) years of mean age, and 0.2 (SD: 0.3) years of duration of diabetes. Males accounted for 59.8% and females accounted for 40.2% of the patients. The overall prevalence of MetS was 66.5% (95% CI: 65.8–67.2%). Participants with MetS were older, more insulin-resistant, and had a larger proportion of males and a smaller proportion of GADA-positive subjects than those without MetS (Table 1).

**Table 1:** Demographic and clinical characteristics of the study patients stratified by metabolic syndrome.

	**Metabolic syndrome**	**Non-metabolic syndrome**	*P*-value^*^	**Effect size**
*n*	10,304	5,188		
Age (year)	52.8 ± 11.6	52.2 ± 11.2	0.001	0.05
Male, *n (%)*	6,384/10,304 (62.0)	2,877/5,188 (55.5)	<0.0001	0.06
South, *n (%)*	6,824/10,304 (66.2)	3,683/5,188 (71.0)	<0.0001	0.05
Educational level				
Tertiary or above, *n (%)*	1,966/9,475 (20.7)	916/4,870 (18.8)	0.006	0.02
Senior high school, *n (%)*	2,412/9,475 (25.5)	1,313/4,870 (27.0)	0.052	0.02
Junior high school or lower, *n (%)*	5,097/9,475 (53.8)	2,641/4,870 (54.2)	0.620	0.00
Current smoking, *n (%)*	3,361/10,189 (33.0)	1,390/5,126 (27.1)	<0.0001	0.06
Current alcohol consumption, *n (%)*	2,067/10,136 (20.4)	739/5,103 (14.5)	<0.0001	0.07
BMI (kg/m^2^)	25.73 ± 3.38	22.67 ± 2.99	<0.0001	0.95
Waist circumference (cm)	91.78 ± 9.67	81.30 ± 8.44	<0.0001	1.15
Systolic BP (mmHg)	131.8 ± 16.2	120.7 ± 14.3	<0.0001	0.73
Diastolic BP (mmHg)	82.4 ± 10.6	75.9 ± 8.9	<0.0001	0.66
TGs (mmol/L) ł	2.14 (1.55–3.19)	1.17 (0.88–1.51)	<0.001	0.51
LDL-C (mmol/L)	2.88 ± 1.00	2.85 ± 0.96	0.097	0.03
HDL-C (mmol/L)	1.09 ± 0.34	1.36 ± 0.37	<0.0001	0.76
HbA1c (%) ł	9.0 (7.2–11.2)	8.9 (6.8–11.6)	0.823	0.01
HOMA2-B (%) ł	46.1 (25.8–73.2)	37.7 (19.4–62.6)	<0.001	0.12
HOMA2-IR ł	1.70 (1.12–2.38)	1.15 (0.71–1.68)	<0.001	0.28
DKA, *n (%)*	578/10,153 (5.7)	371/5,136 (7.2)	0.0002	0.03
GADA positivity, *n (%)*	321/10,304 (3.1)	464/5,188 (8.9)	<0.0001	0.13
Family history of diabetes, *n (%)*	2,832/10,119 (28.0)	1,442/5,115 (28.2)	0.791	0.00
Use of insulin treatment, *n (%)*	2,255/10,277 (21.9)	1,392/5,166 (26.9)	<0.0001	0.06
Use of antihypertensive agents, *n (%)*	3,058/10,277 (29.8)	452/5,166 (8.7)	<0.0001	0.24
Use of lipid-lowering agents, *n (%)*	1,336/10,277 (13.0)	388/5,166 (7.5)	<0.0001	0.08
Types of diabetes				
Type 1, *n (%)*	108 (1.0)	208 (4.0)	<0.0001	0.10
Type 2, *n (%)*	9,936 (96.4)	4,653 (89.7)	<0.0001	0.14
LADA, *n (%)*	260 (2.5)	327 (6.3)	<0.0001	0.09

*BP, blood pressure; DKA, diabetic ketoacidosis; data were presented as number (%) for categorial variables, and mean ± SD or median (interquartile range) for continuous variables*.

* *P-value: for continuous variables, estimates were based on Student t-test for normally-distributed data, or Mann-Whitney U test for highly-skewed data with the mark (ł); for categorical variables, estimates were based on χ^2^ test or Fisher's exact test. For a Student t-test the Effect Size (Cohen's d) Calculator has been chosen. For the Mann-Whitney test an effect size can be achieved by dividing the Z-value with the square root of total number. For categorical data, the effect size is calculated by dividing chi-square value with the total number, and then take the square root*.

### Types of Diabetes and OR of MetS

The prevalence of MetS was 34.2% in T1DM, 44.3% in LADA and 68.1% in T2DM (*P* < 0.05 for comparison of any pairs of them). Using LADA as the referent group, patients with T2DM had higher odds of MetS (OR: 2.69, 95% CI: 2.27–3.17), while patients with T1DM had lower odds of MetS (OR: 0.65, 95% CI: 0.49–0.87) (Model 1, Table 2). The decreased risk of MetS in T1DM (OR: 0.68, 95% CI: 0.50–0.92) and increased risk of MetS in T2DM (OR: 2.82, 95% CI: 2.36–3.37) persisted after adjustment for age, gender, region, education attainment, family history of diabetes, smoking and alcohol consumption habits (Model 2, Table 2). After further adjustment for HOMA2-IR, T2DM remained to be associated with significantly increased risk of MetS (OR: 2.13, 95% CI: 1.76–2.57). On the other hand, the decreased risk of MetS in T1DM was no longer significant (OR: 0.96, 95% CI: 0.70–1.33) (Model 3, Table 2).

**Table 2:** Odds ratio of clinical factors for MetS vs. non-MetS in Chinese newly diagnosed diabetes.

	Model 1^*^		Model 2^*^		Model 3^*^	
	**Crude OR (95% CI)**	***P*-value**	**OR (95% CI)**	***P*-value**	**OR (95% CI)**	***P*-value**
Types of diabetes		<0.0001		<0.0001		<0.0001
Type 2	2.69 (2.27–3.17)	<0.0001	2.82 (2.36–3.37)	<0.0001	2.13 (1.76–2.57)	<0.0001
Type 1	0.65 (0.49–0.87)	0.003	0.68 (0.50–0.92)	0.012	0.96 (0.70–1.33)	0.815
LADA	Reference		Reference		Reference	
Age, year	1.01 (1.00–1.01)	0.001	1.01 (1.00–1.01)	0.002	1.01 (1.00–1.01)	0.012
Male vs. female	1.31 (1.22–1.40)	<0.0001	1.19 (1.09–1.29)	0.0001	1.29 (1.18–1.40)	<0.0001
South vs. North	0.80 (0.75–0.86)	<0.0001	0.81 (0.75–0.88)	<0.0001	0.90 (0.83–0.98)	0.019
Educational level		0.011		0.013		0.005
Tertiary or above	1.11 (1.02–1.22)	0.023	1.02 (0.93–1.13)	0.667	0.97 (0.87–1.08)	0.542
Senior high school	0.95 (0.88–1.03)	0.238	0.89 (0.82–0.97)	0.009	0.86 (0.78–0.94)	0.001
Junior high school or lower	Reference		Reference		Reference	
Family history of diabetes	0.99 (0.92–1.07)	0.791	–		–	
Current smoking	1.32 (1.23–1.43)	<0.0001	1.12 (1.01–1.23)	0.025	–	
Current alcohol consumption	1.51 (1.38–1.66)	<0.0001	1.34 (1.209–1.49)	<0.0001	1.36 (1.22–1.52)	<0.0001
HOMA2-IR		<0.0001	NA			<0.0001
Upper quartile	5.04 (4.52–5.61)	<0.0001	NA		4.68 (4.16–5.25)	<0.0001
Mid-high quartile	2.85 (2.58–3.14)	<0.0001	NA		2.67 (2.40–2.97)	<0.0001
Mid-low quartile	1.54 (1.41–1.69)	<0.0001	NA		1.40 (1.32–1.61)	<0.0001
Bottom quartile	Reference		NA		Reference	

*CI, confidence interval; OR, odds ratio; NA, not applicable*.

* *Model 1 was from univariable analysis. Model 2 was adjusted for age, gender, region, education attainment, family history of diabetes, smoking, and alcohol consumption as selected by forward stepwise (P = 0.05 for entry and P = 0.10 for exclusion). Model 3 was further adjusted for HOMA2-IR*.

### Other Determinants of MetS in Newly Diagnosed Diabetes

In addition to types of diabetes, MetS was also independently associated with older age (OR: 1.01, 95% CI: 1.00–1.01), male gender (OR: 1.29, 95% CI: 1.18–1.40) and alcohol consumption (OR: 1.36, 95% CI: 1.22–1.52). Also, the OR of the bottom vs. top quartiles of HOMA2-IR for MetS was statistically significant (OR: 4.68, 95% CI: 4.16–5.25). Residence in the South (OR: 0.90, 95% CI: 0.83–0.98) and senior high school education attainment (OR: 0.86, 95% CI: 0.78–0.94) were associated with lower risks of MetS (Model 3, Table 2).

### Composition of MetS by Types of Diabetes

For different types of diabetes, the most frequent combinations were hypertension + elevated waist circumference in T2DM, hypertension + elevated waist circumference + hypertriglyceridemia in LADA, and hypertriglyceridemia + low HDL-C in T1DM. With respect to the frequencies of individual features of the MetS, hypertension was the most common abnormality regardless of types of diabetes, while an elevated waist circumference was least frequently seen in T1DM (Table 3).

**Table 3:** Number and percentage of patients with metabolic abnormalities by type of diabetes.

	**T1DM**	**LADA**	**T2DM**
*n*	316	587	14,589
Waist circumference ≥90 cm in male and 85 cm in female, n (%)^*^	71 (22.5)	186 (31.7)	7,332 (50.3)
Systolic/Diastolic BP ≥130/85 mmHg, n (%)^*^	111 (35.1)	281 (47.9)	8,741 (59.9)
TGs ≥1.7 mmol/L, n (%)^*^	88 (27.8)	199 (33.9)	7,516 (51.5)
HDL-C <1.04 mmol/L, n (%)^*^	106 (33.5)	172 (29.3)	5,569 (38.2)
Metabolic syndrome, n (%)^*^	108 (34.2)	260 (44.3)	9,936 (68.1)
HOMA2-IR	0.25 (0.09–0.52)	1.01 (0.53–1.67)	1.53 (1.00–2.20)

*T1DM, type 1 diabetes mellitus; LADA, latent autoimmune diabetes in adults; T2DM, type 2 diabetes mellitus; BP, blood pressure*.

* *Data of patients with parameters above the cut-point defined by CDS metabolic syndrome criteria were expressed as number (%); HOMA2-IR were expressed as median (interquartile range)*.

## Discussion

Our study generated novel findings that MetS was highly prevalent in newly diagnosed diabetes in China, with approximately two-thirds in T2DM and more than one-third in autoimmune diabetes. Patients with T2DM had the highest risk of MetS, and the increased risk could not be entirely attributable to the severity of insulin resistance and other risk factors. On the other hand, patients with LADA also had a higher risk of MetS than those with T1DM, but the increased risk in LADA was attributable to increased insulin resistance.

Several small studies had reported the prevalence of MetS in T2DM, LADA and T1DM in different populations, predominantly in Caucasians and Hispanics (9, 23). In a Spain study (*n* = 640), the prevalence of MetS was 67.2% in T2DM, 37.3% in LADA and 15.5% in T1DM (23). Asians have a higher body fat percentage and are more prone to central obesity and insulin resistance than their western counterparts at a given age, sex, and BMI (24). Furthermore, inadequacy of compensatory insulin secretion, which could not increase in proportion with the severity of insulin resistance, is prominent in Asian diabetic population (24). Asian population differed from Caucasians also in genetics, diet and lifestyles (24, 25), limiting the extrapolation of the aforementioned findings to Asians. However, there is still a lack of relevant studies conducted throughout the entire diabetes spectrum in Chinese population with large sample size and geographical diversity. In a small sample of patients with newly diagnosed diabetes (*n* = 588), our group found that the prevalence of the National Cholesterol Education Program-Adult Treatment Panel III (NCEP-ATP III) criteria-defined MetS was 10.8% in T1DM, 62.4% in LADA and 69.5% in T2DM (26). In this large nation-wide multicenter survey, we updated the prevalence of MetS to be 34.2% in T1DM, 44.3% in LADA and 68.1% in T2DM. The prevalence of MetS in our survey was similar to that reported in the Action LADA study in Europe (9). Differently, the prevalence of MetS in T1DM in our survey was much higher than the 10.1% in T1DM reported in a previous Chinese survey (*n* = 849) (27). Given that the prevalence of MetS increases with increasing age (28), and the much younger age of T1DM patients in that Chinese survey (the median age: 22 years) (27), the observed high prevalence of MetS in our patients is not at odds with the findings of that Chinese survey. Using age at diagnosis ≥30 years as one of the inclusion criteria and the CDS criteria for definition of MetS, which was more appropriate for Chinese population for cardiovascular risk assessment (18), our survey generated a more reliable estimation of the prevalence of MetS in newly diagnosed diabetes. Had we adopted the NCEP-ATP III criteria for diagnosis of MetS, the prevalence of MetS was 38.6% in T1DM, 50.6% in LADA, 74.1% in T2DM.

In addition to types of diabetes, our survey also found that older age, male gender, residence in the South, senior high school education attainment, alcohol consumption, and higher HOMA2-IR were all associated with MetS in newly diagnosed diabetes. Previous studies found that older age (28) and higher HOMA-IR (7) were associated with higher risks of MetS in diabetes, while residence in the South (29) and a relatively high education level (15) were associated with lower risks of MetS, in support of our findings. However, we did not find an association between smoking and MetS, inconsistent with other studies conducted in the general population (30). Also, our findings that alcohol consumption and male gender were associated with increased odds ratio of MetS, were in contrast to the literature (29, 31). In this regard, moderate alcohol consumption proved to be cardiovascular-protective (32). Because our definition of alcohol drinkers was more likely to include heavy drinkers and misclassify mild-to-moderate drinkers to non-drinkers, our findings regarding the association between alcohol consumption and MetS risk were more likely driven by heavy drinking. Types of alcoholic beverages were not documented in our study and further studies are needed to address or confirm types, frequency and quantity of alcohol consumption in their relationships with increased risk of MetS. Regarding the gender-specific difference in the risk for MetS, there was still no unequivocal conclusion, and numerous studies reported that MetS risk in women was not inferior to that in men (9, 29). Indeed, females are genetically more insulin resistant than males (33). However, a complex interplay of sex hormones, metabolic cytokines, environmental factors and lifestyles on cardiovascular profiles might confound the observed risk (34, 35). Moreover, the presence of overt diabetes might considerably attenuate the female-male gradient (36). The use of different criteria for MetS further contributed to the inconsistency among studies.

MetS is a strong risk factor for T2DM and the increased prevalence of MetS may be one of the causes for rapid secular increase in the prevalence of T2DM in China (37). It can be expected that there was a high prevalence of MetS in patients with T2DM. It is of interest to observe that the prevalence of MetS was also quite high in LADA and T1DM in our and other populations. In this regard, the Action LADA study reported that the increased risk of MetS in autoimmune diabetes, i.e., T1DM and LADA, as compared to non-diabetic controls, was attributable to hyperglycemia, but the increased risk of MetS in T2DM was not. The authors of the study suggested that autoimmune diabetes was not due to decreased insulin sensitivity (9). It is established that insulin resistance in LADA, albeit less severe than in T2DM, was more pronounced than in T1DM (38). Similar to T2DM, studies also showed that sweetened beverage intake, overweight/obesity and physical inactivity were strongly predictive of LADA (39, 40). In this connection, our study found that LADA had a higher risk of MetS than T1DM and the increased MetS risk in LADA was due to increased insulin resistance. Because MetS greatly increases the risk of cardiovascular disease in both the general population (1) and the diabetic population (41–43), our findings support use of insulin sensitizers in LADA. Our study also confirms that T2DM was associated with markedly higher risk of MetS, independent of traditional risk factors and even insulin resistance, supporting multicausality of cardiovascular disease in T2DM. Indeed, apart from decreased insulin sensitivity, many other abnormalities such as endothelial stress, chronic low-grade systemic inflammation and hypercoagulation were common in both T2DM and MetS, and these abnormalities intertwined to form a complex network of causality for macro- and micro-vascular diseases in T2DM (44).

Our study has clinical and public health implications. A focus on individual components of MetS, namely hyperglycemia, hypertension, obesity and dyslipidemia separately, is routine in clinical practice, while MetS as an entity is unaddressed given the lack of evidence at population level, especially in Chinese, and our study filled the gap. The findings indicate that newly-diagnosed diabetic population in China are facing an epidemic of MetS, and thus, clinicians should pay more attention to the cardiometabolic profiles of diabetic patients and develop targeted strategies against the risk factors of MetS. Furthermore, although our previous pilot study showed rosiglitazone might have a beneficial effect on beta cell function of LADA patients (45), the roles of insulin sensitizers on the long-term cardiovascular outcomes are largely unknown. Our results suggest that insulin resistance accounted for the residual MetS risk in LADA patients. Future prospective cohort studies are warranted to confirm whether MetS and MetS components are associated with increased risks of diabetes complications, and randomized controlled trials may also be needed if the findings were confirmed by prospective cohorts, to test the efficacy and effectiveness of insulin sensitizers in the management of LADA for potential cardiovascular benefits.

Our study had several strengths. First, our study was a large multi-center study with subjects recruited from tertiary care hospitals across the seven geographic regions of China. Thus, this study had a good representativeness of patients with newly diagnosed adult-onset diabetes in China. Second, the antibody assay was performed in a single well-equipped laboratory, which ensured high quality measurement of the antibody and etiologic classification of diabetes. Our study also had limitations. First, we only used GADA titers to define LADA and some of the “true LADA” patients may be misclassified as T2DM patients. However, negative GADA only represented <5% of the total LADA patients (46), so major bias due to misclassification of LADA was unlikely. Second, socio-economic status was not collected in our survey. Third, the participating hospitals used different C-peptide assay kits although the same method was used. This may have led to a large variance in C-peptide concentrations. However, the participating hospitals were all tertiary care center with good quality control measures in operation. Potential bias due to the non-standardization should be small if any. Finally, our study was a cross-sectional survey and the associations reported by us were not necessarily suggesting causal relationships. Longitudinal studies are needed to examine whether and how the prevalence of MetS by types of diabetes changes over time.

## Conclusions

In summary, our study found that MetS was highly prevalent, up to 66.5% in newly-diagnosed diabetes, with the risks being the highest in T2DM and the second highest in LADA. The increased risk of MetS in T2DM could not be explained by traditional risk factors of MetS and increased insulin resistance. However, the higher risk of MetS in LADA vs. T1DM was attributable to increased insulin resistance in LADA. These findings support use of insulin sensitizers in LADA for management of CVD risk factors. Besides, age, gender, geographical residence, education attainment, alcohol consumption and HOMA2-IR were independent determinants of MetS. Further prospective cohort studies are needed to test whether targeted strategies against these risk factors could lower the Mets rates in diabetes.

## Data Availability Statement

The ethical approval obtained for this study prevents the human data being shared publicly to protect patients' privacy. Requests to access the datasets should be directed to ZZ (zhouzhiguang@csu.edu.cn). This would be passed to the ethics committee who will decide whether they can access the data directly.

## Ethics Statement

The study was reviewed and approved by the ethics review committee of the Second Xiangya Hospital, Central South University [No. (2014) 32], and ethics review committee/institutional review board of each participating hospital. The participants provided their written informed consent before recruitment.

## Author Contributions

ZZ, XL, XYang, and CC designed the study. XYang and CC analyzed the data. XL, CC, XT, XYan, HZ, JL, and LJ collected serum samples and clinical data. XL and CC wrote the manuscript. XL, XYang, and ZZ reviewed and edited the manuscript. All authors critically revised the manuscript for important intellectual content and approved the final manuscript.

### Conflict of Interest

The authors declare that the research was conducted in the absence of any commercial or financial relationships that could be construed as a potential conflict of interest.

## References

[1] O'Neill S O'Driscoll L. Metabolic syndrome: a closer look at the growing epidemic and its associated pathologies. Obes. Rev. (2015) 16:1–12. 10.1111/obr.12229 25407540

[2] DeBoer MD Filipp SL Gurka MJ. Use of a metabolic syndrome severity Z score to track risk during treatment of prediabetes: an analysis of the diabetes prevention program. Diabetes Care. (2018) 41:2421–30. 10.2337/dc18-1079 30275282 PMC6196828

[3] Fourlanos S Dotta F Greenbaum CJ Palmer JP Rolandsson O Colman PG. Latent autoimmune diabetes in adults (LADA) should be less latent. Diabetologia. (2005) 48:2206–12. 10.1007/s00125-005-1960-7 16193284

[4] Carlsson A Sundkvist G Groop L Tuomi T. Insulin and glucagon secretion in patients with slowly progressing autoimmune diabetes (LADA). J Clin Endocrinol Metab. (2000) 85:76–80. 10.1210/jc.85.1.76 10634367

[5] Yang L Zhou ZG Huang G Ouyang LL Li X Yan X. Six-year follow-up of pancreatic beta cell function in adults with latent autoimmune diabetes. World J Gastroenterol. (2005) 11:2900–5. 10.3748/wjg.v11.i19.2900 15902725 PMC4305656

[6] Xiang YF Zhao YJ Zhou ZG. Latent autoimmune diabetes in adults: evidences for diabetes spectrum? Chin Med J. (2013) 126:783–8. 23422206

[7] Alexander CM Landsman PB Teutsch SM Haffner SM. NCEP-defined metabolic syndrome, diabetes, and prevalence of coronary heart disease among NHANES III participants age 50 years and older. Diabetes. (2003) 52:1210–4. 10.2337/diabetes.52.5.1210 12716754

[8] Bingley PJ Mahon JL Gale EA. Insulin resistance and progression to type 1 diabetes in the European Nicotinamide Diabetes Intervention Trial (ENDIT). Diabetes Care. (2008) 31:146–50. 10.2337/dc07-0103 17959864

[9] Hawa MI Thivolet C Mauricio D Alemanno I Cipponeri E Collier D. Metabolic syndrome and autoimmune diabetes: action LADA 3. Diabetes Care. (2009) 32:160–4. 10.2337/dc08-1419 18945926 PMC2606853

[10] Diabetes Control and Complications Trial (DCCT)/Epidemiology of Diabetes Interventions and Complications (EDIC) Study Research Group Intensive diabetes treatment and cardiovascular outcomes in type 1 diabetes: the DCCT/EDIC study 30-year follow-up. Diabetes Care. (2016) 39:686–93. 10.2337/dc15-1990 26861924 PMC4839174

[11] Orchard TJ Forrest KY Kuller LH Becker DJ. Lipid and blood pressure treatment goals for type 1 diabetes: 10-year incidence data from the Pittsburgh Epidemiology of Diabetes Complications Study. Diabetes Care. (2001) 24:1053–9. 10.2337/diacare.24.6.1053 11375370

[12] Edwards KL Hutter CM Wan JY Kim H Monks SA. Genome-wide linkage scan for the metabolic syndrome: the GENNID study. Obesity. (2008) 16:1596–601. 10.1038/oby.2008.236 18421265

[13] Zhou Z Xiang Y Ji L Jia W Ning G Huang G. Frequency, immunogenetics, and clinical characteristics of latent autoimmune diabetes in China (LADA China study): a nationwide, multicenter, clinic-based cross-sectional study. Diabetes. (2013) 62:543–50. 10.2337/db12-0207 23086039 PMC3554388

[14] WHO expert consultation Appropriate body-mass index for Asian populations and its implications for policy and intervention strategies. Lancet. (2004) 363:157–63. 10.1016/S0140-6736(03)15268-3 14726171

[15] Lan Y Mai Z Zhou S Liu Y Li S Zhao Z. Prevalence of metabolic syndrome in China: an up-dated cross-sectional study. PLoS ONE. (2018) 13:e196012. 10.1371/journal.pone.0196012 29668762 PMC5906019

[16] Garralda-Del-Villar M Carlos-Chilleron S Diaz-Gutierrez J Ruiz-Canela M Gea A Martinez-Gonzalez MA. Healthy lifestyle and incidence of metabolic syndrome in the SUN cohort. Nutrients. (2018) 11:65. 10.3390/nu11010065 30598006 PMC6357191

[17] Alberti KG Zimmet PZ. Definition, diagnosis and classification of diabetes mellitus and its complications. Part 1: diagnosis and classification of diabetes mellitus provisional report of a WHO consultation. Diabetes Med. (1998) 15:539–53. 9686693 10.1002/(SICI)1096-9136(199807)15:7<539::AID-DIA668>3.0.CO;2-S

[18] Chinese Diabetes Society Guidelines for prevention and treatment of type 2 diabetes in Chinese (2017 edition). Chin J Diabetes Mellitus. (2018) 10:4–67. 10.3760/cma.j.issn.1674-5809.2018.01.003

[19] Levy JC Matthews DR Hermans MP. Correct homeostasis model assessment (HOMA) evaluation uses the computer program. Diabetes Care. (1998) 21:2191–2. 10.2337/diacare.21.12.2191 9839117

[20] Huang G Xiang Y Pan L Li X Luo S Zhou Z. Zinc transporter 8 autoantibody (ZnT8A) could help differentiate latent autoimmune diabetes in adults (LADA) from phenotypic type 2 diabetes mellitus. Diabetes Metab Res Rev. (2013) 29:363–8. 10.1002/dmrr.2396 23390067

[21] Sullivan GM Feinn R. Using Effect Size-or Why the P Value Is Not Enough. J Grad Med Edu. (2012) 4:279–82. 10.4300/JGME-D-12-00156.1 23997866 PMC3444174

[22] Pautz N Olivier B Steyn F. The use of nonparametric effect sizes in single study musculoskeletal physiotherapy research: a practical primer. Phys Ther Sport. (2018) 33:117–24. 10.1016/j.ptsp.2018.07.009 30077090

[23] Mollo A Hernandez M Marsal JR Esquerda A Rius F Blanco-Vaca F. Latent autoimmune diabetes in adults is perched between type 1 and type 2: evidence from adults in one region of Spain. Diabetes Metab Res Rev. (2013) 29:446–51. 10.1002/dmrr.2411 23483713

[24] Chan JC Malik V Jia W Kadowaki T Yajnik CS Yoon KH. Diabetes in Asia: epidemiology, risk factors, and pathophysiology. JAMA. (2009) 301:2129–40. 10.1001/jama.2009.726 19470990

[25] Zhu M Xu K Chen Y Gu Y Zhang M Luo F. Identification of novel T1D risk loci and their association with age and islet function at diagnosis in autoantibody-positive T1D individuals: based on a two-stage genome-wide association study. Diabetes Care. (2019) 42:1414–21. 10.2337/dc18-2023 31152121

[26] Xiang Y Zhou P Li X Huang G Liu Z Xu A. Heterogeneity of altered cytokine levels across the clinical spectrum of diabetes in China. Diabetes Care. (2011) 34:1639–41. 10.2337/dc11-0039 21617097 PMC3120206

[27] Huo L Ji L Deng W Shaw JE Zhang P Zhao F. Age distribution and metabolic disorders in people with Type 1 diabetes in Beijing and Shantou, China: a cross-sectional study. Diabetes Med. (2018) 35:721–8. 10.1111/dme.13616 29512926

[28] Thorn LM Forsblom C Fagerudd J Thomas MC Pettersson-Fernholm K Saraheimo M. Metabolic syndrome in type 1 diabetes: association with diabetic nephropathy and glycemic control (the FinnDiane study). Diabetes Care. (2005) 28:2019–24. 10.2337/diacare.28.8.2019 16043748

[29] Gu D Reynolds K Wu X Chen J Duan X Reynolds RF. Prevalence of the metabolic syndrome and overweight among adults in China. Lancet. (2005) 365:1398–405. 10.1016/S0140-6736(05)66375-1 15836888

[30] Slagter SN van Vliet-Ostaptchouk JV Vonk JM Boezen HM Dullaart RP Kobold AC. Associations between smoking, components of metabolic syndrome and lipoprotein particle size. BMC Med. (2013) 11:195. 10.1186/1741-7015-11-195 24228807 PMC3766075

[31] Freiberg MS Cabral HJ Heeren TC Vasan RS Curtis ER. Alcohol consumption and the prevalence of the Metabolic Syndrome in the US.: a cross-sectional analysis of data from the Third National Health and Nutrition Examination Survey. Diabetes Care. (2004) 27:2954–9. 10.2337/diacare.27.12.2954 15562213

[32] Gazzieri D Trevisani M Tarantini F Bechi P Masotti G Gensini GF. Ethanol dilates coronary arteries and increases coronary flow via transient receptor potential vanilloid 1 and calcitonin gene-related peptide. Cardiovasc Res. (2006) 70:589–99. 10.1016/j.cardiores.2006.02.027 16579978

[33] Han LL Wang YX Li J Zhang XL Bian C Wang H. Gender differences in associations of serum ferritin and diabetes, metabolic syndrome, and obesity in the China Health and Nutrition Survey. Mol Nutr Food Res. (2014) 58:2189–95. 10.1002/mnfr.201400088 25163435 PMC6636645

[34] Auer MK Ebert T Pietzner M Defreyne J Fuss J Stalla GK. Effects of sex hormone treatment on the metabolic syndrome in transgender individuals: focus on metabolic cytokines. J Clin Endocrinol Metab. (2018) 103:790–802. 10.1210/jc.2017-01559 29216353

[35] Zhu Z Wu F Lu Y Wu C Wang Z Zang J. Total and nonheme dietary iron intake is associated with metabolic syndrome and its components in chinese men and women. Nutrients. (2018) 10:1663. 10.3390/nu10111663 30400363 PMC6266186

[36] Pucci G Alcidi R Tap L Battista F Mattace-Raso F Schillaci G. Sex- and gender-related prevalence, cardiovascular risk and therapeutic approach in metabolic syndrome: a review of the literature. Pharmacol Res. (2017) 120:34–42. 10.1016/j.phrs.2017.03.008 28300617

[37] Yang W Lu J Weng J Jia W Ji L Xiao J Prevalence of diabetes among men and women in China. N Engl J Med. (2010) 362:1090–101. 10.1056/NEJMoa0908292 20335585

[38] Buzzetti R Zampetti S Maddaloni E. Adult-onset autoimmune diabetes: current knowledge and implications for management. Nat Rev Endocrinol. (2017) 13:674–86. 10.1038/nrendo.2017.99 28885622

[39] Lofvenborg JE Andersson T Carlsson PO Dorkhan M Groop L Martinell M. Sweetened beverage intake and risk of latent autoimmune diabetes in adults (LADA) and type 2 diabetes. Eur J Endocrinol. (2016) 175:605–14. 10.1530/EJE-16-0376 27926472

[40] Carlsson S Midthjell K Tesfamarian MY Grill V. Age, overweight and physical inactivity increase the risk of latent autoimmune diabetes in adults: results from the Nord-Trondelag health study. Diabetologia. (2007) 50:55–8. 10.1007/s00125-006-0518-7 17096113

[41] Thorn LM Forsblom C Waden J Saraheimo M Tolonen N Hietala K. Metabolic syndrome as a risk factor for cardiovascular disease, mortality, and progression of diabetic nephropathy in type 1 diabetes. Diabetes Care. (2009) 32:950–2. 10.2337/dc08-2022 19196885 PMC2671127

[42] Bonora E Targher G Formentini G Calcaterra F Lombardi S Marini F. The Metabolic Syndrome is an independent predictor of cardiovascular disease in Type 2 diabetic subjects. Prospective data from the Verona Diabetes Complications Study. Diabetes Med. (2004) 21:52–8. 10.1046/j.1464-5491.2003.01068.x 14706054

[43] Isomaa B Almgren P Tuomi T Forsen B Lahti K Nissen M. Cardiovascular morbidity and mortality associated with the metabolic syndrome. Diabetes Care. (2001) 24:683–9. 10.2337/diacare.24.4.683 11315831

[44] Grandl G Wolfrum C. Hemostasis, endothelial stress, inflammation, and the metabolic syndrome. Semin Immunopathol. (2018) 40:215–24. 10.1007/s00281-017-0666-5 29209827 PMC5809518

[45] Zhou Z Li X Huang G Peng J Yang L Yan X. Rosiglitazone combined with insulin preserves islet beta cell function in adult-onset latent autoimmune diabetes (LADA). Diabetes Metab Res Rev. (2005) 21:203–8. 10.1002/dmrr.503 15386806

[46] Shi X Huang G Wang Y Liu Z Deng C Li X. Tetraspanin 7 autoantibodies predict progressive decline of beta cell function in individuals with LADA. Diabetologia. (2019) 62:399–407. 10.1007/s00125-018-4799-4 30594957

